# Social Context–Induced Song Variation Affects Female Behavior and Gene Expression

**DOI:** 10.1371/journal.pbio.0060062

**Published:** 2008-03-18

**Authors:** Sarah C Woolley, Allison J Doupe

**Affiliations:** 1 Department of Psychiatry, University of California, San Francisco, San Francisco, California, United States of America; 2 Department of Physiology, University of California, San Francisco, San Francisco, California, United States of America; University of Washington, United States of America

## Abstract

Social cues modulate the performance of communicative behaviors in a range of species, including humans, and such changes can make the communication signal more salient. In songbirds, males use song to attract females, and song organization can differ depending on the audience to which a male sings. For example, male zebra finches (Taeniopygia guttata) change their songs in subtle ways when singing to a female (directed song) compared with when they sing in isolation (undirected song), and some of these changes depend on altered neural activity from a specialized forebrain-basal ganglia circuit, the anterior forebrain pathway (AFP). In particular, variable activity in the AFP during undirected song is thought to actively enable syllable variability, whereas the lower and less-variable AFP firing during directed singing is associated with more stereotyped song. Consequently, directed song has been suggested to reflect a “performance” state, and undirected song a form of vocal motor “exploration.” However, this hypothesis predicts that directed–undirected song differences, despite their subtlety, should matter to female zebra finches, which is a question that has not been investigated. We tested female preferences for this natural variation in song in a behavioral approach assay, and we found that both mated and socially naive females could discriminate between directed and undirected song—and strongly preferred directed song. These preferences, which appeared to reflect attention especially to aspects of song variability controlled by the AFP, were enhanced by experience, as they were strongest for mated females responding to their mate's directed songs. We then measured neural activity using expression of the immediate early gene product ZENK, and found that social context and song familiarity differentially modulated the number of ZENK-expressing cells in telencephalic auditory areas. Specifically, the number of ZENK-expressing cells in the caudomedial mesopallium (CMM) was most affected by whether a song was directed or undirected, whereas the caudomedial nidopallium (NCM) was most affected by whether a song was familiar or unfamiliar. Together these data demonstrate that females detect and prefer the features of directed song and suggest that high-level auditory areas including the CMM are involved in this social perception.

## Author Summary

Vocal communication in many species, including humans, is affected by social cues. In the zebra finch, for example, males make subtle changes to the length, tempo, and variability of their courtship songs (directed songs) relative to songs performed in isolation (undirected songs). Using a behavioral approach assay, we found that female zebra finches strongly prefer the sound of directed over undirected song. Interestingly, female preferences were influenced by the variability of note pitch, showing stronger preferences for directed songs when they were less variable in pitch than the undirected songs. Pitch variability is controlled by a forebrain–basal ganglia circuit, which may represent a neural substrate on which selection acts to shape behavior. Preference for directed song was also increased when the singer was familiar to the listener, suggesting that song preferences are enhanced by experience. Based on the expression of an immediate early gene associated with memory formation and plasticity, we found that two high-level auditory areas were differentially responsive to the category of song females heard, with one area responding to whether songs were directed or undirected, and a second area to whether songs were familiar or unfamiliar. Together, these data demonstrate that females detect and prefer the male's changed performance during courtship singing and suggest that neurons in high-level auditory areas are involved in this social perception.

## Abstract 3

Female songbirds are attentive to subtle changes in male song, particularly to the variability of pitch. High-level auditory areas may generate females' behavioral preferences.

## Introduction

Across species and sensory modalities, associations between signal production and reception are critical for intraspecific communication, and receivers' preferences and biases can shape signal characteristics [1]. In addition to driving ultimate changes in signal characteristics, receivers can also have immediate influence on signal production, and the use of different signals or alteration of signals depending on the audience has been seen in a range of species including chickens [2,3], frogs [4], lizards [5], dolphins [6], humans [7,8], and songbirds [9–15]. With respect to within-individual variation in acoustic behavior, animals have most often been observed to alter the overall amount of sound or the frequency of occurrence of particular components such as “sexy” syllables [4,11,12,14]. For some of these changes, it has also been demonstrated that females, the intended audience for these signals, display preferences for the altered signals [16–22]. A particularly striking example of social context–dependent vocal change comes from humans, who alter not the amount or identity of utterances, but rather the pitch, intonation contours, and cadence of the speech they direct toward infants [7,8]. Infants have been shown to prefer this infant directed speech over adult-directed speech [23,24], and characteristics of infant-directed speech promote language learning [25]. Zebra finch males provide another such example, changing not only the quantity of their song [13] in response to females, but also its vocal quality. Specifically, when singing to females (directed song), they sing faster [13,26,27] and produce syllables with subtly but significantly less spectral variability [27–29] than when they sing alone (undirected song).

An advantage of studying zebra finches is that the neural basis of some of these changes in song is known. In particular, social context–dependent changes in the spectral variability of song syllables depend on alterations in activity of a specialized forebrain–basal ganglia circuit known as the anterior forebrain pathway (AFP). The earliest indication of this came from the discovery of dramatic differences in immediate early gene (IEG) expression in the AFP during directed and undirected singing, which suggested that undirected song might reflect “practice” and ongoing adult learning, whereas directed song was focused on courtship [30]. The large changes in IEG expression were surprising, given the minimal context-dependent differences in song, and led to the speculation that small differences in the song might be salient to females [30]. Subsequent studies of the outflow nucleus of the AFP using neurophysiology, stimulation, and lesions, in combination with detailed song analysis, showed that the AFP rapidly switches between firing states that actively enable song syllable variability—during undirected song—or that are associated with increased song stereotypy—in courting birds [27,31,32]. Because of this, it has been proposed that variable undirected song reflects a less aroused state, in which the more-variable firing pattern in the AFP enables motor “exploration” that is important for learning and plasticity, whereas the stereotyped directed song reflects a highly motivated or “performance” state, which is associated with more stable pallial–basal ganglia circuit activity [27,30,32]. Activity in both normal and diseased mammalian basal ganglia may similarly be associated with behavioral plasticity versus stereotypy [33–36]. However, a pivotal aspect of the exploration versus performance hypothesis—that female zebra finches attend to directed–undirected differences in song and show a preference for the putative performance state—has not been tested.

We addressed this question by using a behavioral approach assay and found that female zebra finches strongly prefer directed over undirected songs despite how relatively subtle the directed–undirected vocal differences are. The preference for directed song was present in sexually naive as well as mated females, but the magnitude of the preference was influenced by song familiarity, with mated females showing the strongest responses to the directed songs of their mate. Moreover, the strength of preference in individual birds was correlated with an acoustic feature of song known to be controlled by the AFP, the spectral variability of syllables.

To look broadly for neuronal populations that might be specifically involved in the females' social discrimination and preference, we used a marker for neuronal activation, the IEG *ZENK* (the avian homologue of *zif268*, *egr-1*, *NGFI-A*, and *Krox24*). *ZENK* and other IEGs in higher telencephalic auditory areas of birds are selectively activated by song playback and show different responses to conspecific song, compared to heterospecific song, as well as to more subtle aspects of song [37–48]. Here we compared the number of ZENK-expressing cells in response to matched amounts of familiar directed and undirected songs, as well as unfamiliar directed songs. In the nucleus mesencephalicus lateralis pars dorsalis (MLd) in the ascending auditory system we found induction of similar numbers of ZENK-expressing cells in response to each of these stimuli, which suggests that in the MLd, the number of ZENK-expressing cells depends simply on the amount of song exposure. However, in two high-level forebrain auditory areas, we found that the number of ZENK-expressing cells differed depending on the song's familiarity and social context. The number of ZENK-expressing cells in the caudal medial nidopallium (NCM), in accord with previous studies [49–51], was most strongly affected by unfamiliar songs, whereas in the caudal medial mesopallium (CMM) the number of ZENK-expressing cells was most strongly affected by directed songs. Thus, consistent with the performance hypothesis, females prefer directed over undirected song, especially features controlled by the songbird pallial–basal ganglia circuit. Moreover, they show a neural correlate of this social preference in a central auditory area.

## Results

### Mated Females Prefer Mate's Directed Song to Both Unfamiliar Conspecific Song and Their Mate's Undirected Song

To test whether females can both discriminate between two stimuli and express a preference for one stimulus type over another, we used a behavioral approach assay (Figure 1A, see Methods for details). In the assay, females were placed between two speakers that alternately broadcast competing stimuli, and the amount of time individuals spend in the chamber near each speaker was recorded. We first confirmed the findings of previous studies [52,53], showing that mated females can discriminate their mate's directed song from the directed song of an unfamiliar conspecific and strongly prefer their mate's song. Examples of familiar and unfamiliar stimuli used in the assay are illustrated in Figure 1B. Each zebra finch song is composed of a series of individual acoustic elements or “syllables,” separated by at least 5 ms of silence, which are sung in a stereotyped order (creating the “motif,” black bars, Figure 1B). Multiple renditions of the motif are sung in succession, preceded by a number of simple repeated introductory notes (white bars, Figure 1B), to form a song or song “bout.” The acoustic structure of syllables as well as their sequencing are learned features of song. Hence, as depicted in Figure 1B, the songs of different males are typically quite distinct and can be discriminated from each other based on a number of acoustic features. We found that females could readily distinguish and strongly preferred their mate's song over the song of an unfamiliar male (Figure 2A; *n* = 18, Wilcoxon signed rank = 68.00, *p* < 0.0001). In all experiments, females were excluded from the analysis if they failed to meet established criteria for demonstrating a preference (see Methods and [52,53] for criteria), which for this experiment meant excluding only two females. All of the remaining 18 females spent significantly more time in the chamber that was broadcasting their mate's directed song than in the chamber broadcasting unfamiliar directed song. These data both confirm previous reports that females can discriminate based on familiarity and indicate that our behavioral assay is effective, at least under conditions where there are very salient acoustic differences between stimuli.

**Figure 1:**
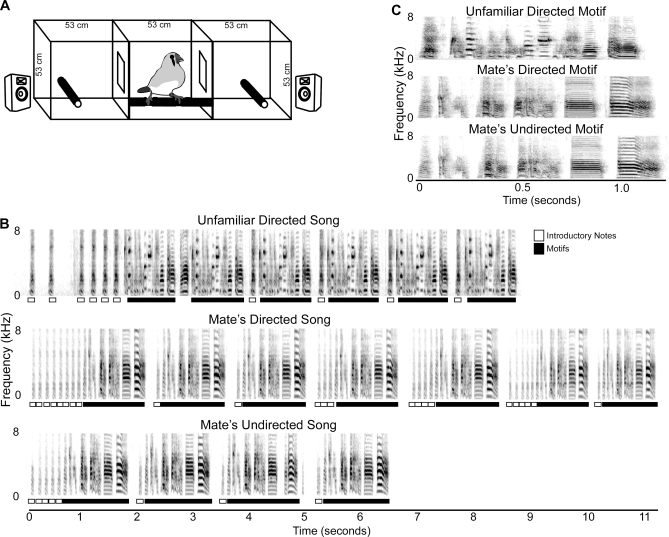
Diagram of Behavioral Assay and Sonograms Illustrating Song Differences between and within Males (A) Diagram of the two-choice behavior assay. Tests began when females were in the center chamber containing food, water, and a perch. Stimuli were played alternately from speakers at each end of the cage, and females moved freely into the side chambers. Thick lines in the side and center chambers indicate perches. (B) Examples of a directed song bout from an unfamiliar male and directed and undirected song bouts from a mate. Introductory notes are indicated with open lines, and motifs are indicated with black lines. In these examples, there are more motifs in both of the directed songs (unfamiliar and mate's song) than the undirected song. (C) Sonograms of single motifs from each of the song examples. These examples highlight the much greater differences between the songs of different birds (e.g., unfamiliar male versus mate) than between songs produced in the two contexts by the same bird (mate's directed versus mate's undirected).

**Figure 2:**
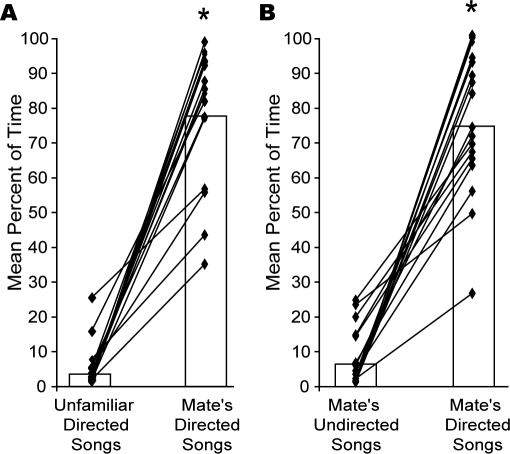
Females Prefer the Directed Songs of Their Mate (A) Mean percent of time spent in the unfamiliar directed and mate's directed song chambers. Individuals are plotted as diamonds. Females spent significantly more time in the mate's directed song chamber (*p < 0.05). (B) Mean percent of time spent in the mate's undirected and directed song chambers. Individuals are plotted as diamonds. Females spent significantly more time in the directed song chamber (*p < 0.05).

We next asked whether females could discriminate between and show preferences for the directed and undirected songs of their mates. Acoustically, such a discrimination is likely to be a much more difficult task than differentiating between the songs of a mate and an unfamiliar male. While the syllables that make up the core motif are often quite different between birds, the structure of the core motif is almost identical in the directed and undirected songs from the same bird (Figure 1C). Moreover, while there are a number of previously characterized differences in the acoustic structure of directed and undirected song [13,26–29], these song features are highly overlapping for individual songs. Despite this acoustic similarity, however, we found that females could readily distinguish between their mate's directed and undirected songs and showed strong preferences for directed song (Figure 2B; *n* = 17, Wilcoxon signed rank = 85.5, *p* < 0.0001). Only three females were excluded from the analysis. All of the remaining 17 mated females spent significantly more time in the chamber broadcasting their mate's directed song than in the chamber broadcasting their mate's undirected song. These data clearly indicate that females attend to differences in the social context of their mate's songs and express a consistent preference for the directed song in our approach assay.

### Mated and Naive Females Prefer Directed over Undirected Song of Unfamiliar Males

We performed two experiments to assess whether experience with a male or his songs was necessary for the preference for directed song. First, we investigated whether mated females detect and prefer generic features that differ between directed and undirected songs, or whether they attend only to specific features present in the songs of their mate. We did this by testing a subset (*n* = 6) of our mated females with unfamiliar directed and undirected songs. Each female was tested with the directed and undirected songs of six different unfamiliar males. All songs were from mated males whose mates had shown a significant preference for their directed song. We found that mated females tested on these unfamiliar songs were influenced by whether the song was directed or undirected. On tests where females showed a response (*n* = 4 responses in four birds), they always preferred directed song (Figure 3A, Student's *t*-test, *t* = −4.51, *p* = 0.011).

**Figure 3:**
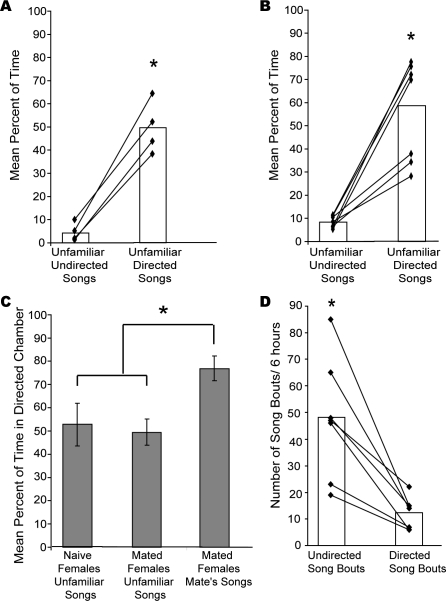
Mated and Naive Females Prefer Unfamiliar Directed Songs to Unfamiliar Undirected Songs (A) Mean percent of time that mated females spent in the unfamiliar undirected and directed song chambers for the four behavior tests on which females displayed a preference. Females significantly preferred unfamiliar directed to undirected songs (*p < 0.05). (B) Mean percent of time that naive females spent in the unfamiliar undirected and directed song chambers. Seven females showed significant preferences for at least one of the stimuli. Because some females responded to stimuli from more than one male, diamonds represent the average response for each female. Naive females significantly preferred directed song to undirected song (*p < 0.05). (C) Mated females tested on their mate's songs spent significantly more time in the directed song chamber (*p < 0.05) than did naive or mated females tested on unfamiliar songs. (D) Number of undirected and directed song bouts produced over a 6-h period by males housed with their mates. While directed song bouts tended to be longer and accompanied by additional courtship behaviors and copulation attempts, males sang significantly more undirected song bouts (*p < 0.05).

Second, we tested sexually naive females (*n* = 8), to determine whether these birds could also differentiate between and show preferences for directed and undirected songs from unfamiliar males. Naive females had never been mated and therefore their experience with directed song was limited to instances during tutoring, between 0 and 60 d, when their father sang song directed to his mate (see Methods). When naive females exhibited a response (*n* = 11 responses in seven birds), they always preferred the directed songs over the undirected songs of unfamiliar males (Figure 3B, Wilcoxon signed rank = 14.00, *p* = 0.016). These data strongly suggest that there are generic context-dependent features of the songs that females can detect and for which they exhibit a differential preference. Further, this discrimination and preference does not rely on other experience such as the paired association of directed song with courtship and copulation interactions with males, and in addition, in the case of naive females, does not require substantial prior experience with directed and undirected songs or mating experience.

Whereas females that responded to unfamiliar stimuli showed a significant preference for directed song, familiarity was generally still an important variable. One demonstration of the importance of familiarity is that mated and naive females tested on unfamiliar songs did not respond to all or even, in a small number of cases, any songs. For example, four mated females each responded to songs from only one of the six males on which they were tested, which means that there were responses on only four of 36 tests. Two mated females did not respond to any of the stimuli. Similarly, there were responses on only 11 of 48 tests of naive females on songs from unfamiliar males, and one of the eight naive females tested did not respond to any of the stimuli.

Moreover, on the tests where females were responsive, there was an effect of song familiarity on the magnitude of the preference for directed song (Figure 3C; Kruskal Wallis test, χ^2^ = 7.25, *p* = 0.007). The amount of time females spent in the directed song chamber was greatest among mated females tested on songs of their mate, and significantly lower for mated (Wilcoxon rank sum, χ^2^ = 3.93, *p* = 0.047 ) and naive females (Wilcoxon rank sum, χ^2^ = 7.25, *p* = 0.007 ) tested on directed and undirected songs from unfamiliar males. These data indicate that, while females tested on unfamiliar songs prefer directed over undirected songs, the degree of preference for directed song is accentuated when females are presented with the familiar songs of their mates.

For the mated females, we were additionally interested in whether the expressed preference for directed songs reflected a greater (or exclusive) exposure to directed songs. We therefore examined the behavior and songs of males singing in the presence of their mates, to determine whether they were exclusively “directed” in nature. In many studies [26,27,29–31], directed song is collected by presenting a female to an isolated male, while undirected song is collected when the male is alone. Using this paradigm, previous studies have found that 85%–100% of the song performed in the presence of a female under these conditions is directed song [29,30]. However, we were interested in whether mated males that have been cohabiting with their mate for months or years perform only directed song, or whether males sing undirected song despite the presence of their mate. To investigate this, we studied eight of our mated pairs, which had been housed together continuously for at least 4 mo prior to video recording, and scored 6-h videotapes of each pair for whether males were singing directed or undirected song. We defined directed song as instances in which the male oriented toward the female and performed at least one of the following courtship behaviors: hopping/dancing, beak wiping, or fluffing of the body feathers while flattening the head feathers. In contrast, undirected song was performed while facing away from the female and without any courtship behaviors [15,30]. Based on these criteria, we found that males housed for extended periods with females performed both directed and undirected song in the presence of their mate. Males sang significantly more bouts of undirected song than directed song (Figure 3D; paired *t*-test, *t* = 4.64, *p* = 0.002). These data confirm the suggestion of [29], based on *ZENK* expression data, that males housed with females sing a mix of directed and undirected song. Thus, we conclude that the preference for directed song among mated females is not simply a preference for the more familiar song, although we cannot rule out that the association of directed song with courtship behavior may be important for the preference.

### Measures of Song Features and Their Association with Female Preferences

We measured a number of aspects of the directed and undirected song stimuli used in our behavioral assay to investigate whether female preferences were correlated with particular acoustic features of song. We focused on features at a number of levels of song organization that had previously been found to differ between directed and undirected song. Specifically, for the five directed and undirected stimuli of each bird that we used in the behavior assay, we quantified the number of introductory notes, number of motifs, and motif tempo [13,26,27], and, as measures of variability, we quantified the coefficient of variation (CV; standard deviation/mean) of the fundamental frequency of syllables containing harmonic stacks [27] and a general measure of overall syllable stereotypy—the “accuracy” score from the Sound Analysis Pro software (SAP, http://ofer.sci.ccny.cuny.edu/html/body_sound_analysis.html [54,28,29]). The directed stimuli had significantly more motifs (Wilcoxon signed rank = 86.00, *p* = 0.001) and introductory notes (Wilcoxon signed rank = 100.50, *p* < 0.0001) than the undirected stimuli, and directed motifs were faster (Wilcoxon signed rank = −80.50, *p* = 0.003). In addition, the variability (measured as the CV) of the fundamental frequency was significantly higher for syllables with harmonic stacks during undirected renditions than during directed renditions (Wilcoxon signed rank = −65.50, *p* = 0.019). Using the general measure of syllable stereotypy from SAP, we did not find significant differences between the directed and undirected stimuli of our birds, perhaps because they were from older and more experienced birds than other samples analyzed with this measure.

We next investigated whether the degree of preference shown by females was correlated with any of the features of the songs that we quantified, in particular whether there was a stronger preference for directed song when there was a large difference between directed and undirected song. We analyzed the relationship between the percent of time spent in the directed chamber and percent difference between directed and undirected songs for each of the features we measured (number of introductory notes, number of motifs, song tempo, CV of the fundamental frequency, and syllable stereotypy). We found that only the percent difference in the CV of the fundamental frequency between directed and undirected song was correlated with the degree of preference (Figure 4A). Specifically, when the directed song was less variable than the undirected song, the preference for directed song was stronger than when the levels of variability were similar, or when directed song was more variable than undirected song (*R* ^2^ = 0.36, *F* = 8.28, *p* = 0.012) .

**Figure 4:**
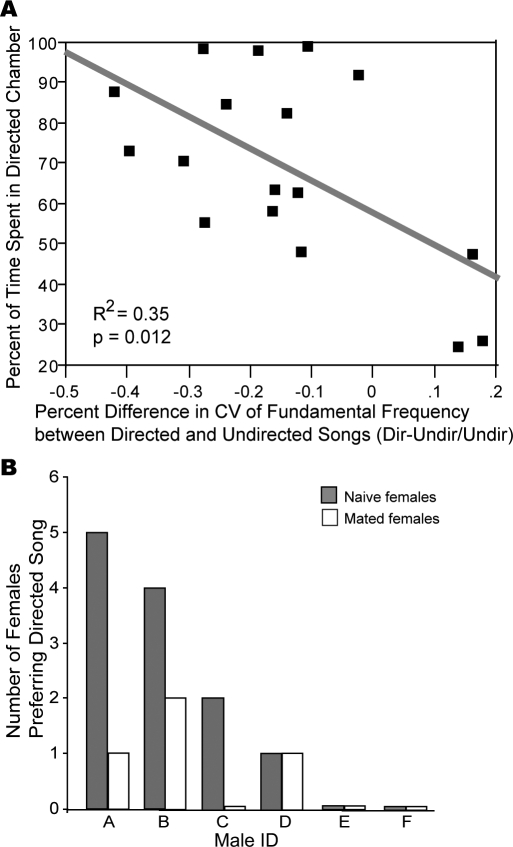
Song Variability and Singer Identity Influence Preferences for Directed Song (A) Plot of the correlation between the percent of time females spent in the directed chamber and the percent difference between the directed and undirected stimuli in the CV of the fundamental frequency of harmonic stacks. When the CV was higher in directed songs than in undirected songs, the preference for directed song was weaker than when the CV was higher in undirected songs. (B) Whether females responded to unfamiliar songs depended on which male the songs came from. Graphed is the number of naive (gray bars) and mated (open bars) females that showed a preference for the directed songs of each of the six unfamiliar males. Overall, there was a significant effect of stimulus male on the responses of naive females (*p* < 0.05) but not a significant effect on the responses of mated females.

We also tested whether the responses of mated and naive females presented with songs from unfamiliar males were associated with any of the song measures we studied. All song bouts used were from mated males whose mates preferred their directed song, indicating that the directed songs of each male can, in principle, be discriminated from the undirected renditions. However, we found that only certain males' songs elicited responses from naive females while others did not (Figure 4B; Chi square test, χ^2^ =15.48, *p* = 0.009). Interestingly, the stimulus sets that were least preferred by naive females were also those that were least preferred by mated females tested on unfamiliar stimuli (e.g., Figure 4B, columns E and F), although the χ^2^ test for mated females was not significant. However, we found no correlations between the degree of preference shown by individual females and any of the song features we measured.

### Auditory Stimuli Differentially Induce ZENK Expression in the NCM, CMM, and MLd

Given that the behavioral responses revealed that females could discriminate between and show preferences for familiar over unfamiliar songs (e.g., Figure 2A) and directed over undirected songs (e.g., Figure 2B), we were interested in whether any of the auditory areas that have previously been implicated in song perception might participate in these processes. To address this issue we quantified the expression of the IEG ZENK by counting ZENK-immunoreactive (ZENK-ir) cells in three auditory areas in response to song playback. We counted cells in a midbrain auditory region, and the presumed avian homologue of the mammalian inferior colliculus, the nucleus mesencephalicus lateralis pars dorsalis (MLd), and in two higher order forebrain auditory areas, the caudal medial nidopallium (NCM) and the caudal medial mesopallium (CMM), in response to matched amounts of their mate's directed song, their mate's undirected song, or the directed song of an unfamiliar conspecific.

For all areas, playback of songs elicited a significant increase in the number of ZENK-expressing cells relative to silent controls (NCM, ANOVA *F* = 189.79, *p* = 0.005; CMM, ANOVA *F* = 94.2, *p* = 0.01; and MLd, ANOVA *F* = 17.94, *p* = 0.052). However, as described below, the induction of ZENK-expressing cells in response to different song stimuli systematically differed across the three brain areas.

#### The number of ZENK expressing cells in the MLd was equally increased by all stimuli.

As a control for the general auditory efficacy of the stimuli, and to investigate whether the number of ZENK-expressing cells in the ascending auditory system was differentially affected by our stimuli, we quantified the number of ZENK-ir cells in the MLd (Figure 5A). In the MLd, the number of ZENK-expressing cells was similar among females that heard unfamiliar directed song, their mate's directed song, or their mate's undirected song (Figure 5B and 5C). Thus, hearing the same amount of song, regardless of the singer or other attributes of the song, induced similar numbers of ZENK-expressing cells in the ascending auditory system.

**Figure 5:**
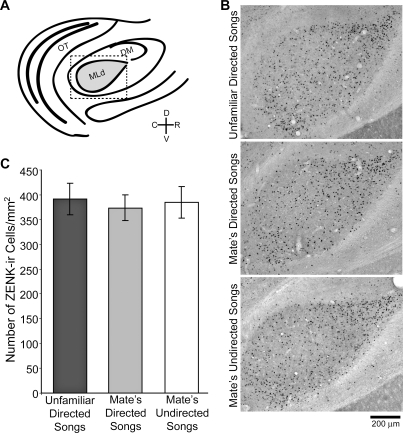
The Number of ZENK-Expressing Cells in the MLd Is Equally Increased by All Stimuli (A) Diagram of the region sampled within the MLd. Dashed line indicates the area shown in the images in (B). DM, dorsomedial nucleus of the intercollicular complex; OT, optic tectum. (B) Representative images of ZENK staining in the MLd in each of the stimulus groups. (C) Mean number of cells per area in the MLd. There were no significant differences between any of the groups in the number of ZENK expressing cells.

#### The number of ZENK-expressing cells in the NCM was preferentially increased by unfamiliar stimuli.

In the NCM, we counted ZENK-ir cells in two regions (caudal and ventral) based on similar studies in zebra finches and other species [43,45] (see Methods; Figure 6A). The number of ZENK-expressing cells was modulated by the type of stimulus in both the caudal (ANOVA *F* = 6.24, *p* = 0.017) and ventral regions (ANOVA *F* = 9.95, *p* = 0.004). In both regions, females that heard directed song from an unfamiliar male had significantly more ZENK expressing cells than females that heard either their mate's directed (Student's t-test; caudal: t=3.05, p=0.012; ventral: t=3.15, p=0.01) or mate's undirected (Student's t-test; caudal: t=3.26, p=0.009; ventral t=4.42, p=0.001) song (Figure 6B, C). There was no difference between females that heard their mate's directed and undirected song in either the caudal or ventral region (Figure 6B, C). Thus, the number of ZENK expressing cells in the NCM is affected by song familiarity, showing the greatest increase in response to unfamiliar songs.

**Figure 6:**
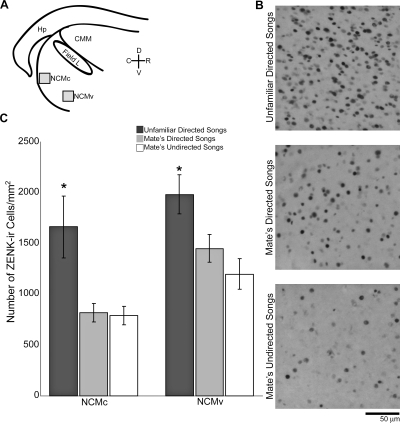
The Number of ZENK-Expressing Cells in the NCM Is Preferentially Increased by Unfamiliar Stimuli (A) Diagram of the regions sampled within NCM. HP, hippocampus; NCMc, caudal region of NCM; NCMv, ventral region of NCM. Both regions were similar to those used in ZENK cell counts in NCM in other studies (see Methods). (B) Representative images of ZENK staining in NCM (caudal region) in each of the stimulus groups. (C) Mean number of cells per area in the caudal and ventral regions of the NCM. Females that heard unfamiliar directed songs had significantly more ZENK-expressing cells than females that heard their mate's directed or undirected songs. (*p < 0.05).

#### The number of ZENK-expressing cells in the CMM was preferentially increased by directed stimuli.

The number of ZENK-expressing cells in the CMM (Figure 7A) was also modulated by song playback (ANOVA *F* = 5.90, *p* = 0.02). Females that heard unfamiliar directed song (Student's *t*-test; *t* = 3.27, *p* = 0.008) or their mate's directed song (Student's *t*-test; *t* = 2.4, *p* = 0.038) had more ZENK-expressing cells than females that heard their mate's undirected song (Figure 7B and 7C). Moreover, there were no differences in the level of ZENK staining between females that heard unfamiliar directed song and their mate's directed song. The number of ZENK-expressing cells in the CMM is therefore affected by the song's social context, with the greatest induction in response to directed song.

**Figure 7:**
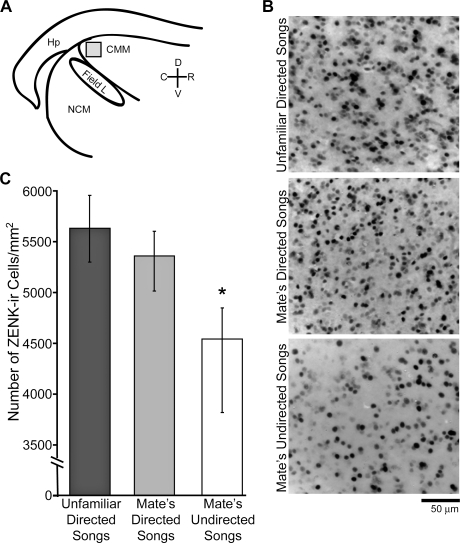
The Number of ZENK-Expressing Cells in the CMM Is Preferentially Increased by Directed Stimuli (A) Diagram of the region sampled within CMM. HP, hippocampus. (B) Representative images of ZENK staining in CMM in each of the stimulus groups. (C) Mean number of cells per area in the CMM. Females that heard their mate's undirected songs had significantly fewer ZENK-expressing cells than females that heard their mate's directed songs or unfamiliar directed songs (*p < 0.05).

## Discussion

In many species that use vocal communication—including songbirds, frogs, and humans—the vocal signal is modulated depending on the audience for which it is performed [2–4,7–15]. Male zebra finches subtly alter their learned song when delivering it to a female, compared to when they sing alone [13,15,26,27]. Here, using a behavioral preference assay in response to natural variation in male song, we asked whether females prefer song produced in a particular social context. We found that females can discriminate between songs from directed and undirected social contexts, and, despite the relative subtlety of directed–undirected song differences, females prefer their mate's directed over undirected songs as strongly as they prefer the songs of their mate to the very different songs of an unfamiliar male. In addition, both mated and naive females preferred unfamiliar directed song to undirected song, although this preference was less strong than that of females for their mate's directed songs. We then investigated neural correlates of the female preferences using a measure of neural activity, the number of cells expressing the IEG product ZENK, in two higher order auditory areas. We found that the number of ZENK-expressing cells was differentially modulated by both the song's familiarity and social context. In the NCM, the number of ZENK-expressing cells was greatest in response to the directed songs of an unfamiliar male, whereas in the CMM, the directed songs of a female's mate and of an unfamiliar male both induced more ZENK-expressing cells than did the mate's undirected song.

Directed–undirected differences in song have been shown to depend, in part, on altered activity in a specialized forebrain-basal ganglia circuit, the AFP [27]. Both neural firing and the expression of plasticity-associated genes in the AFP switch between patterns of activity that are associated with syllable stereotypy, during directed song, and patterns of activity associated with syllable variability, during undirected song [30,31]. This has led to the hypothesis that males can rapidly shift between undirected “motor exploration” and directed “performance” states [27,30]. The demonstration that female birds can detect the alteration in song, and prefer the less-variable directed version, supports the idea that the stereotyped directed songs of males reflect a highly motivated, active performance state, aimed at the female, and accompanied by less changeable pallial–basal ganglia circuit firing.

In zebra finches, the subtle but consistent differences between directed and undirected song occur at multiple levels of song organization. Directed songs tend to have a faster tempo and more motifs and introductory notes than undirected songs [13,26,27] and to contain syllables with more stereotyped and reproducible structure across renditions [27–29]. Strikingly, we found that the behavioral preference for directed over undirected song was correlated not with tempo or motif number, but with the degree of context-dependent difference in variability of syllable structure, specifically with the CV of the fundamental frequency of syllables with harmonic stacks. The latter difference has been shown to depend on the integrity of the projections from the AFP to the song motor pathway, whereas other context-dependent differences, such as motif number, do not [27]. The female's preference for the features of song actively controlled by the AFP provides further evidence for the importance of this circuit's contribution to song, and for the AFP's role in switching between stereotyped “performance” and more-variable states. In addition, these data imply that the female nervous system is sensitive to song variability, an effect that highlights the utility of presenting multiple examples of entire song bouts rather than single songs or motifs. However, because females did not completely reverse their preference on the occasions in which there was more variability in the directed songs, syllable variability is likely not the only feature females attend to in assessing song quality. Rather, while the other features we measured were not independently correlated with the behavioral preference for directed song, one possibility is that such features may be used simultaneously or in combination during song evaluation. Additional studies focused on manipulation of syllable variability as well as other song features that show context-dependent differences will be key to understanding how females weigh various features when determining the attractiveness of a song.

Both mated and naive females preferred unfamiliar directed song to undirected song in our assay, indicating that auditory signals alone must carry information about generic directed–undirected differences that females can detect. However, because the responses of females to unfamiliar songs were less robust than the responses of females to their mate's songs, and because many unfamiliar songs failed to elicit any response, there are clearly additional factors that modulate the strength of directed song preference. One possibility is that females assessing an unfamiliar male normally rely on other cues in addition to song, such as courtship dancing or beak and feather color [55–58]. Mated females may also have developed stronger song preferences as a result of associations between directed song and other visual cues or courtship behaviors that accompany directed song. Finally, experience with the songs themselves may also be important. Mated females have had multiple occasions to hear their mate's directed and undirected songs, giving them ample opportunity to learn the finer differences between songs from the two contexts. However, mating alone does not appear to create a preference for all directed songs, because mated females, like naive females, respond less strongly to directed songs from unfamiliar males.

Just as the behavior of females was affected by song familiarity and social context, the number of ZENK-expressing cells in response to song playback was also affected by these two behavioral axes, but differentially in two separate brains areas. In the NCM, the number of ZENK-expressing cells was affected primarily by song familiarity, which is in accord with studies in zebra finches and other species [42,47–51]. Because our study included familiar directed and undirected songs as well as unfamiliar songs, but the number of ZENK-ir cells showed no differentiation between the two types of familiar songs, the data suggest that the number of ZENK-expressing cells in the caudal and ventral regions of the NCM specifically differentiates the categories “unfamiliar” and “familiar,” regardless of social context. In contrast to those in the NCM, we found that the number of ZENK-expressing cells in the CMM was differentially affected by directed and undirected song, irrespective of song familiarity. The number of ZENK-expressing cells in the CMM was higher in females that heard directed song, regardless of whether it was from their mate or from an unfamiliar conspecific, than in those that heard their mate's undirected song. This supports the idea that CMM is sensitive to the categories “directed” and “undirected,” independently of song familiarity. This contrasts with previous work where either song attractiveness or a combination of attractiveness and familiarity were manipulated, making it difficult to discern whether CMM responds to song familiarity, attractiveness, or both [43–45]. At the behavioral level, females responded to both singer familiarity and social context simultaneously. Based on our neural data, we cannot determine whether the interaction between song familiarity and social context seen behaviorally occurs within either the NCM or CMM in a manner not apparent as a change in ZENK expression, or whether the information emerges through convergent output of the NCM and CMM. However, in either case, these data highlight the regional divergence in the neural processing of song along these two behavioral axes, and the importance of considering both axes in understanding the role of the NCM and CMM in song perception.

In previous studies of ZENK expression in the CMM, the number of ZENK-expressing cells was higher for behaviorally preferred, but quite dissimilar songs [43,44]. We found that the CMM can make distinctions even among highly similar familiar stimuli. Male zebra finches sing the same core motif in both social conditions but alter the composition of the song bout, for example the number of motifs and introductory notes, the tempo, and the stereotypy of the fine structure of particular syllables [13,26–29]. Although we controlled for the total amount of song heard in the different groups (see Methods), we did so by altering the amount of silence between individual stimuli, not by altering the stimuli themselves. Thus, we preserved the natural differences in tempo, stereotypy, fine structure of syllables, and bout structure. That the CMM shows a differential response to directed and undirected song implies that it may be attuned to these subtle differences in song and syllable structure. Further, because the number of ZENK-expressing cells was high in the CMM for both mate's directed song and unfamiliar conspecific directed song, it seems likely that the increase in ZENK is due to particular acoustic features of directed song rather than associations with other aspects of courtship that often accompany directed singing. Thus, the CMM may be involved not just in the recognition of a song's social context, but in song quality evaluation, including that involved in mate selection.

The NCM has previously been shown to respond to song familiarity, with novel songs inducing high levels of ZENK [50], while in response to familiar stimuli, ZENK expression habituates or is low [47,49–51]. However, in prior studies in which there was a significant difference in the number of ZENK-expressing cells in the NCM, songs were rendered familiar either through minutes or days of passive playback and ZENK responses were tested, at most, 24 h after the song was last heard [47,49–51]. In our study, songs were familiar because of months or years of interactions between birds, and ZENK responses were measured at least 2 wk, and in some cases up to 1 mo, after the song was last heard. Thus, our data indicate that the “habituation” of the ZENK response in NCM to a familiar song is both long-lasting and socially relevant. The mammalian homolog of ZENK, zif268 [59], has been implicated in synaptic plasticity as well as memory acquisition, consolidation, and retrieval based on studies in rats and mice [60–64]. Our data support the hypothesis [42,47,48,50] that ZENK activity in the NCM may be causally related to the learning or consolidation of new information, including the formation of long-term memories associated with novel songs.

There are multiple ways in which the differential ZENK expression in the NCM and CMM could arise. One possibility is that the NCM and CMM serve as auditory filters, specifically attending to features present in novel and familiar songs or directed and undirected songs. Alternatively, these brain areas may belong to networks of “social processing” nuclei, like those found in mammals, which include cortical sensory areas as well as amygdala, striatum, and other forebrain regions [65,66]. Activity in the NCM and CMM could be regulated secondarily by modulatory inputs from other social brain structures, which might themselves be sensitive to differences in song familiarity or social context. For example, both the NCM and CMM receive noradrenergic projections that may modulate the processing of auditory stimuli [67]. Further investigation of the activity of single neurons in the CMM and NCM in response to songs that differ in their degree of familiarity, social context, or variability as well as in response to neuromodulators will help elucidate how the auditory forebrain processes complex, behaviorally relevant stimuli and how processing is affected by social experience.

## Methods

### Subjects.

Mated females (*n* = 20, >180 d old) were paired with males for at least 3 mo and in some cases for up to 3 y. All pairs were producing fertile eggs at the time of behavior testing, and 15 of the pairs had previously raised at least one clutch of offspring. Females were separated from their mates for at least 4 d but no more than 1 wk prior to behavior testing. Naive females (*n* = 8, >120 d old) were sexually naive at the time of testing. Naive females were raised with parents and siblings until 60 d of age. During this time, they likely heard both their father's undirected song as well as his directed song when he sang to his mate. At 60 d of age, females were moved into group cages containing two–eight similarly aged females. Group cages were maintained in our colony with opaque plastic dividers between all cages, allowing auditory but not visual interaction with neighboring cages. At least 1 wk prior to testing, naive females were moved from the colony to individual cages in sound-attenuating chambers (Acoustic Systems) containing up to three other individually housed females. All animals were housed on a 14:10 light:dark cycle with finch seed, mash, grit and water provided ad libitum. In addition, animals were given egg food, lettuce, and millet once per week. Procedures adhered to Institutional Animal Care and Use Committee–approved protocols and National Institutes of Health guidelines for the care and use of animals.

### Behavior testing.

Behavior tests were modeled after [52]. On the day of testing, females were placed in a three-chambered cage (each chamber was 53 × 53 × 53 cm for a total size of 159 × 53 × 53 cm) with food, water, and a perch in the center chamber (Figure 1A). On each end of the center chamber, an opening (32 × 32 cm) led into the adjacent chamber where a perch was located three-quarters of the way between the doorway and the far end of the chamber. Speakers (SuperZero, NHT) powered by an amplifier (CE 1000, Crown Audio) were located outside the cage at each end. A camera (KPC S700C, PalmVID) and microphone (IsoMax B3P4FF25B, Countryman Associates) connected to preamplifier (302 dual microphone preamp, Symetrix) a video monitor and video cassette recorder (VCR; SR V10U , JVC) were located above the cage such that all three chambers were visible on the monitor. The cage and speakers were housed within a Plexiglas box and surrounded on four sides by acoustic foam.

For every test, females were placed in the testing chamber and given at least 1.5 h to acclimate. Testing started when the female was located in the center chamber. A single stimulus was then played from the first speaker, followed by a single stimulus from the second speaker. Stimuli were played alternately from each speaker, one stimulus at a time, for 30 min. There were five stimuli, all from the same male, assigned to each speaker, which were presented multiple times in random order over the course of the 30 min test. The interval between each presentation was at least 1 s longer than the length of the longest stimulus used in the test (range: 10–25 s). After a female completed one 30-min test, she was tested again, at least 1 h later, with the stimulus set assigned to each speaker reversed. Thus, for each type of stimulus, females were given a pair of tests to ensure that they were moving into a chamber based on the stimuli and not because of a bias for a particular region of the apparatus. The female's position and other behaviors (such as copulation solicitation displays) were recorded on the VCR. Videotapes of all choice tests were scored by an observer blind to the stimuli assigned to each speaker. For all tests, the time at which a female moved into a different chamber was recorded.

All mated females received pairs of tests on two types of stimuli. First, each female was assayed for her response to her mate's directed song versus the directed song of an unfamiliar male conspecific, and second, she was assayed for her response to her mate's directed song versus her mate's undirected song. A subset of mated females (*n* = 6) were further assayed for their responses to the directed and undirected songs of six unfamiliar conspecific males. Naive females were tested for their responses to the unfamiliar directed and undirected songs from the same six unfamiliar males. All six unfamiliar males were mated, and the behavioral responses of their mates had previously been investigated. In a single day, each female received at most three pairs of tests, with at least 1 h between each individual test.

### Stimuli.

Stimuli were chosen from a repertoire of the songs of the mates of the 20 mated females recorded over a period of 2 d to 1 wk. Males were separated from their mates and housed individually in a small cage in a sound isolation chamber containing a microphone and video camera connected to a video monitor and VCR. The output from the microphone was filtered from 0.3–10 kHz (Model 3382 Filter, Krohn-Hite) and digitally recorded using a song-activated recording system [A. Leonardo (California Institute of Technology, Pasadena, California) and C. Roddey (University of California San Francisco, San Francisco, California)]. For all males, we recorded at least 15 bouts each of directed and undirected song. Undirected song was recorded when the male sang spontaneously while alone. For directed song, a small cage containing a muted female, which was not the male's mate, was placed in the soundbox, and the male was viewed on the video monitor to determine whether he performed directed song. Only instances where males oriented toward the female and displayed at least one of the following components of courtship were considered directed singing: fluffing of the body feathers while flattening feathers on top of the head, hopping/dancing, and beak wiping [15,30]. Females were muted by placing a small piece of plastic tubing in their bronchi. Briefly, females were anesthetized using equithesin and a small incision was made in the skin and air sac to expose the syrinx. An incision was made along the midline of the syrinx and a small piece of plastic tubing was placed into each bronchia. The syrinx incision was closed using Nexaband (Abbott Animal Health) and the skin was closed with suture.

Song was analyzed off-line using software written in the Matlab programming language (Mathworks). We defined a bout of song as an epoch of continuous sound that contained periods of silence less than 800 ms. For each song bout, we determined syllable boundaries for all syllables using an automated, amplitude-based segmentation algorithm. Each syllable was then given an identifying label used to determine the syllable sequence within a motif as well as the number of introductory notes, call notes, complete motifs, and incomplete motifs in all of the song bouts (see Figure 1 for examples of song bouts). Based on these measures, we selected five examples both of directed and undirected song bouts from each male's repertoire to be used as stimuli. We chose five renditions rather than just single exemplars in order to represent directed and undirected song as a class and expose females to the range and variation in the number of motifs, introductory notes, tempo, and syllable structure present in a male's repertoire. Thus, we included two songs that represented the range of bout compositions (greatest and least bout duration) and three that were representative of the mean bout length. If males had more variable syntax, examples of both the most common syntax as well as variants of the prototypical syntax were included as stimuli. Call notes that preceded introductory notes or followed the termination of the song bout were not included. Songs with significant noise artifacts (e.g., cage noises) were avoided. All songs were normalized by their maximum amplitude and converted to wav files (sampling frequency 44.1 kHz).

### Analysis.

All statistics were performed using JMP software version 3.2.2 (SAS Institute) for the PC. Unless otherwise noted, we used an alpha value of *p* < 0.05.

*Behavior tests:* In contrast to operant conditioning paradigms, the approach assay that we used tests for self-generated preferences, and no additional reward is provided by the experimental setup. Consequently, females are not required to demonstrate a preference for the stimuli presented. While many females displayed clear preferences by choosing to spend a majority of their time on the side of a particular stimulus (e.g., directed song) during both tests, we found that females could also fail to demonstrate a preference. Therefore, we established a criterion for what constituted a preference based on previous phonotaxis studies in zebra finches [52,53]. Specifically, we required that females spend at least twice as much time on the side of one stimulus as on the other, on both tests, and spend a minimum of 3 min (10% of the total testing time) in either of the non-center chambers. Thus, we excluded females who were unresponsive either because they did not spend sufficient amounts of time in the non-center chamber or because they had a side bias, as well as undecided females who spent equal amounts of time in the two sound chambers. In practice, using these criteria we found that a very high percentage of mated females responded to the songs of their mate.

For all pairs of tests in which females responded, we determined the amount of time spent in each stimulus chamber on both behavior tests and then calculated the average percent of the total time in the apparatus that was spent in the chamber for a particular stimulus category (e.g., directed or undirected song). Naive and mated females assayed on songs from unfamiliar males occasionally responded to songs from more than one male, in which case we calculated an average percent of total time for each female for all males to which she responded. For all comparisons (mate's directed versus unfamiliar directed, mate's directed versus mate's undirected, unfamiliar directed versus unfamiliar undirected), we calculated the percent difference in the amount of time spent in each chamber (% time spent in directed chamber – % time spent in undirected chamber/% time spent in the directed chamber + % time in undirected chamber). We tested these distributions for normality using a Shapiro-Wilk *W* test and performed Student's *t*-tests on the percent difference when distributions were normally distributed and nonparametric Wilcoxon signed rank tests when the data were not normally distributed. For clarity, all data are plotted as the actual percent of time spent in each chamber.

We also wanted to investigate whether preferences were affected by stimulus familiarity by directly comparing the responses of females from the three different stimulus groups: mated females tested on their mate's songs, mated females tested on unfamiliar songs, and naive females tested on unfamiliar songs. We focused on tests in which females responded and compared the percent of time females in each of the three groups spent in the directed and undirected chambers. We performed a Kruskal Wallis rank sum test on the percent of time females from each group (mated females tested on their mate's songs, mated females tested on unfamiliar songs, and naive females tested on unfamiliar songs) spent in the directed chamber. In the event that the overall test was significant, we performed pairwise Wilcoxon rank sum tests between each of the three groups.

*Song stimuli:* We quantified differences between directed and undirected songs at a number of levels of the song organization based on differences that have previously been described in zebra finches [13,26–29]. For each of the five directed and five undirected songs that were used as stimuli, we determined syllable boundaries for all syllables using an automated, amplitude-based segmentation algorithm and then gave each syllable an identifying label. At the level of song bouts, we counted the number of introductory notes and motifs in each stimulus and calculated the mean number of introductory notes and motifs for each context. We also measured the tempo of the motif from the onset of the first non-introductory note syllable to the onset of the last syllable. We used syllable onsets, because the syllable onset boundaries are sharper and better defined than syllable offsets. We calculated the mean motif tempo across all motifs in the five stimuli for each context.

We also measured the stereotypy of single syllables. As a general measure of syllable stereotypy, we used Sound Analysis Pro 1.04 software [54] to calculate a local similarity score (“accuracy”) as described in [28,29]. Briefly, for each syllable in a male's motif for which we had at least ten examples, we selected ten examples of the syllable (range: 1–3 examples of each syllable from each of the five stimuli) and calculated a score for each syllable example based on measurements of the frequency modulation, wiener entropy, pitch, goodness of pitch, and amplitude modulation. Then we performed 45 pairwise comparisons between the measurements of each of the ten examples to generate a similarity score for each syllable. Within each male, we then averaged the similarity scores for all comparisons for all the syllables we measured for the directed and undirected songs.

We also examined the variability of the fundamental frequency for all syllables with harmonic stacks as described in [27]. Briefly, for a particular syllable, we calculated the autocorrelation of a segment of the sound waveform that had clear harmonic structure. The fundamental frequency was defined as the distance, in Hz, between the zero-offset peak and the highest peak in the autocorrelation function. To verify that we consistently measured the same portion of each syllable, and to ensure that syllables were free of sound artifacts, we visually screened each example of a syllable. We performed a parabolic interpolation of the peak of the autocorrelation function to improve resolution of the frequency estimates [68]. We calculated the fundamental frequency for a minimum of 15 renditions in each behavioral context, and from these measurements, we calculated the mean, standard deviation, and coefficient of variation (CV; standard deviation/mean) of the fundamental frequency. We use the CV of the fundamental frequency as a measure of syllable variability.

For each of these five measurements (number of introductory notes, number of motifs, motif tempo, fundamental frequency CV, syllable stereotypy), we calculated the mean percent difference between directed song and undirected song (directed song – undirected song/undirected song). We performed Wilcoxon signed-rank tests on the directed–undirected difference for each measure to determine whether each song feature differed between the two contexts.

In addition, we investigated whether the degree of preference of mated females for their mate's directed and undirected songs were correlated with any of the song features. We performed a regression of the absolute percent of time females spent in the directed chamber with the percent difference between directed and undirected song, for each song feature. We also investigated whether there were preferences for the songs of particular males among the naive and mated females tested on unfamiliar stimuli using a least-likelihood (χ^2^) test.

### Effects of auditory stimuli on induction of ZENK protein expression.

*Song playback:* All females in the study of ZENK protein expression were mated and had previously received behavior tests. Song playback for ZENK began at least 2 wk, and in some cases as long as 1 mo, after females were separated from their mates. Females were placed in an empty soundbox with a video camera, microphone, and speaker. Food and water were provided ad libitum. Between 3.5–4 h later, the lights were turned off. After 45 min of darkness, females were exposed to 30 min of auditory stimuli. Females heard either their mate's directed song (*n* = 7), their mate's undirected song (*n* = 7), or the directed song of an unfamiliar conspecific (*n* = 5). Each stimulus set consisted of five different renditions of song bouts, all from the same male, that were played in random order for 30 min. The amplitude for each stimulus was normalized to its maximum amplitude and played at a mean sound pressure level of 65 dB (range of 55–75 dB). The interval between each stimulus was set such that all females were exposed to the same total duration of song (11 min of song during 30 min of playback) regardless of which stimulus set they heard. To ensure that we were getting sufficient ZENK induction to song playback, we performed a pilot study in which females that heard unfamiliar directed song were compared to silence controls that did not hear playback. Silence controls were mated females that had not received behavior tests but were isolated and had the lights turned off at the same times as females played unfamiliar directed songs.

*Immunohistochemistry:* One h after the last stimulus was played, females were anesthetized with Isoflurane and perfused with 0.4% heparinized lactated ringers solution followed by 4% paraformaldehyde. The heads were removed, post-fixed in 4% paraformaldehyde for 12 h, and then the brain was removed from the skull and cryoprotected overnight in 30% sucrose. Three sets of 40-μm free-floating sections were cut on a freezing microtome into 0.025 M phosphate buffered saline (PBS). Every third section was used for ZENK immunohistochemistry (IHC). We performed ZENK IHC in three batches with all stimulus groups represented in each batch. All testing and stimulus playback was performed immediately prior to perfusion and staining for all individuals within a batch.

ZENK IHC was similar to that described for FOS IHC by [69]. All steps were performed at room temperature. Briefly, sections were washed in Tris–phosphate buffer (TPBS; 0.01 M Tris in 0.01 M PB and 0.9% saline, with 0.05% Thimerosal) containing 0.3% Triton X-100 (Triton) and 1% normal goat serum (NGS; Antibodies, Inc.) incubated overnight in 5% NGS diluted in TPBS with 0.3% Triton for 1 h and then in rabbit polyclonal anti-Egr1 (Santa Cruz Biotechnology, Egr-1(588) lot # G107; 1:10,000 diluted in TPBS containing 0.3% Triton and 1% NGS). Sections were incubated in goat anti-rabbit secondary antibody (1:200; Vector laboratories) diluted in TPBS containing 0.3% Triton and 1% NGS for 1 h, and then in avidin-biotin complex (Vectastain, Vector Laboratories) in TPBS containing 0.3% Triton. Peroxidase was visualized using 3′3 diaminobenzidine (DAB; Sigma) with nickel intensification, and glucose oxidase to generate hydrogen peroxide. Sections were mounted and dried onto gelatin-coated slides and coverslipped. Adjacent sections were mounted onto slides and stained with cresyl violet. Controls for binding specificity of the antibody, including pre-absorption with immunizing peptide and Western analysis have been previously documented [39].

*Cell counting and analysis:* In all areas, sections were imaged using a Nikon Eclipse E800 microscope and Zeiss Axiocam MRc5 camera connected to a Windows XP computer. We adjusted the microscope for Kohler illumination using a 10× objective and maintained the light level at those settings for all images collected for a particular nucleus.

We counted cells in a midbrain auditory area, the MLd, and in two higher auditory areas in the telencephalon, the NCM and CMM. Images were captured at 10× in the NCM, 20× in the CMM, and 10× in the MLd, saved as JPEGS using Zeiss AxioVision software, then converted to grayscale in Adobe Photoshop. In the MLd, we counted ZENK-ir cells on four sections medial to and including the most lateral section on which the MLd is teardrop shaped (Figure 5A). On each section, we outlined the MLd and measured both the number of ZENK-ir cells and the area of the nucleus to calculate the density of ZENK-ir cells/mm^2^. In the NCM, two regions were cropped from a larger image for analysis. One was located in the most caudal part of the dorsal half of the NCM, near to the most ventral part of the hippocampus (NCMc; Figure 6A). The second region was in the most ventral portion of the NCM (NCMv). We sampled a 200 × 200 μm region from each of these areas and saved them at a higher resolution (of 300 pixels/inch). In the CMM we sampled a 344 × 158 μm region in the most caudal area dorsal to the mesopallial lamina and ventral to the lateral ventricle (Figure 7A). For both the CMM and the NCM, we collected images from every third 40-μm section between 320 and 700 μm from the midline. These regions of both the NCM and CMM were chosen for analysis based on studies of ZENK protein expression in response to auditory stimuli reported in a number of species including zebra finches [43,70], starlings [41,45,71,72], and sparrows [73,74].

For all regions, the number of cells was counted using a computer-assisted image analysis system (Image J from NIH Image) by an observer blind to the experimental treatments. The imaging system assigns each pixel of the digitized image a gray-scale value from 1 to 255. Average pixel density for each digitized image is determined, and the range and frequency of pixel densities are plotted as a histogram. The program divides the pixels into “objects” and “background” and computes a threshold for staining intensity that is just greater than the average of the objects and background (composite average). We measured the threshold for staining intensity for all sections within a nucleus for each individual. Based on those values, for each individual we chose a maximum threshold for staining intensity that was 10%–20% above the automatic value. In general, these values were quite similar between individuals and even similar across IHC groups. Within each nucleus we also set a minimum and maximum size (in pixels) for what could be considered an object based on the size of cells in the nucleus at a particular magnification.

*Statistical analyses:* In the MLd, we performed a two-way ANOVA with stimulus, IHC batch, and stimulus × IHC batch as the independent variables and the mean number of ZENK-positive cells/mm^2^ for all sections as the dependent variable. To compare between stimuli when there was an overall effect of stimulus, we performed Student's *t*-tests. In the NCM and CMM, we initially performed a multivariate ANOVA with stimulus, IHC batch, and brain region (caudal NCM, ventral NCM, and CMM), and the interaction terms as independent variables and the mean number of ZENK-positive cells/mm^2^ for all sections in the caudal NCM, ventral NCM, and CMM regions as the dependent variables. We found an overall interaction between stimulus, IHC, and brain region for NCM and CMM (MANOVA, *F* = 2.54, *p* = 0.049). We therefore looked at the number of ZENK-expressing cells in the caudal NCM, dorsal NCM, and CMM separately using ANOVAs for each brain region with stimulus, IHC batch, and stimulus × IHC batch as the independent variables and the mean number of ZENK-positive cells/mm^2^ as the dependent variable. To compare between stimuli when there was an overall effect of stimulus, we performed Student's *t*-tests.

## Abbreviations

AFP: anterior forebrain pathway
CMM: caudomedial mesopallium
CV: coefficient of variation
IEG: immediate early gene
IHC: immunohistochemistry
MLd: nucleus mesencephalicus lateralis pars dorsalis
NCM: caudomedial nidopallium
